# Assisted peritoneal dialysis: a feasible KRT modality for frail older patients with end-stage kidney disease (ESKD)

**DOI:** 10.1038/s41598-021-94032-8

**Published:** 2021-07-22

**Authors:** Qianhui Song, Hao Yan, Zanzhe Yu, Zhenyuan Li, Jiangzi Yuan, Zhaohui Ni, Wei Fang

**Affiliations:** 1 Department of Nephrology, Renji Hospital, School of Medicine, Shanghai Jiao Tong University, No. 160, Pujian Road, Pudong District, Shanghai, 200127 People’s Republic of China; 2 Shanghai Center for Peritoneal Dialysis Research, Shanghai, China

**Keywords:** Geriatrics, Outcomes research, Nephrology, Renal replacement therapy

## Abstract

Assisted PD is used as an alternative option for the growing group of frail, older ESKD patients unable to perform their own PD. This study was undertaken to investigate the outcomes of assisted PD in older patients by comparing assisted PD patients with self-care PD patients. This study included all patients aged 70 and above who started on PD in our hospital from 2009 to 2018. Patients were followed up until death, PD cessation or to the end of the study (December 31, 2019). Risk factors associated with mortality, peritonitis and technique failure were evaluated using both cause-specific hazards and subdistribution hazards models. 180 patients were enrolled, including 106 (58.9%) males with a median age of 77.5 (77.2–81.2) years. Among the 180 patients, 62 patients (34.4%) were assisted. Patients on assisted PD group were older, more likely to be female, more prevalent in DM and CVD, with a higher Charlson score than patients undergoing self-care PD (*P* all < 0.05). In the multivariable analysis, assisted patients had a comparable patient survival and peritonitis-free survival compared to self-care PD patients either in the Cox or in the FG models. According to a Cox model, the use of assisted PD was associated with a lower risk of technique failure (cs-HR 0.20, 95% CI 0.04–0.76), but the association lost its statistical significance in the Fine and Gray model. Our results suggest that assisted PD could be a safe and effective KRT modality for older ESKD patients who need assistance.

## Introduction

With the aging of the general population, the number of older individuals developing end-stage kidney disease (ESKD) continues to rise worldwide, accompanied by a much greater demand for kidney replacement therapy (KRT) among the older^1,2^. There was no common consensus to deliver either HD or PD to the older ESKD patients, and several studies suggested comparable or better outcomes with PD among older patients^3–6^. Compared with in-center hemodialysis, peritoneal dialysis offers many potential benefits to older patients, such as less intervention in lifestyle, no need for vascular access, fewer hemodynamic variations and cost-effective, etc^7^. When life expectancy is perceived to be short, quality of life (QoL) may be the priority for older patients, especially the “older elderly”. PD has also been shown to be associated with better quality of life (QoL) and higher satisfaction with treatment^3,5,8^. However, barriers to self-care PD including multimorbidity, physical disabilities and psychosocial problems often emerged with increasing age^9,10^. Assisted PD is defined as PD treatment performed at the patient’s home and with the assistance of a family member, a partner, a community nurse or a healthcare technician^11^. As a feasible option for patients who cannot perform their own PD exchanges, assisted PD have been developed in many countries with the aim of overcoming barriers in older and non-self-sufficient patients, and some studies suggested that the use of assisted PD could increase the utilization of PD among older patients^12–14^. However, whether assisted PD achieved similar outcomes to self-care peritoneal dialysis still remained controversial^15^. Therefore, we conducted the present study to investigate the outcomes of assisted PD in ageing patients, by comparing patients undergoing assisted PD with those on self-care PD in a cohort of older patients.

## Materials and methods

### Patients

In our study, the cut-off for the definition of an ‘older’ individual was 70 years of age. All incident patients aged 70 and above who started on PD between 1 January 2009 and 31 December 2018 at Renji Hospital, Shanghai Jiao Tong University School of Medicine, China, were screened for eligibility. Patients had history of maintenance HD/transplantation, withdrew from PD within 3 months or with incomplete data were excluded from the study. All enrolled patients were dialyzed using lactate-buffered glucose-based PD solutions (Dianeal^®^, Baxter) with twin-bag system. Patients and their caregivers had received standard training after catheterization by PD dedicated nurses^16^. The study was approved by the Human Research Ethics Committee of Renji Hospital, Shanghai Jiao Tong University School of Medicine. All individual information was securely protected and was made available to only the investigators.

### Demographic and laboratory data

The demographic characteristics collected at baseline included age, gender, height, weight, underlying cause of ESKD and comorbid condition status such as diabetes mellitus (DM) and cardiovascular disease (CVD). Hypertension and diabetes were defined either as a comorbid disease or as the etiology of ESKD. CVD was defined as a previous history of any following condition: acute coronary syndrome, heart failure, cerebral infarction or hemorrhage, coronary artery atherosclerosis confirmed by percutaneous coronary intervention (PCI) or coronary artery bypass grafting (CABG) therapy. The Charlson comorbidity index was adopted to reflect the burden of comorbid conditions. Body mass index (BMI) was calculated as the weight (kg) divided by the square of height in meters (BMI = weight [kg]/height [m^2^]).

Baseline laboratory parameters included hemoglobin, serum albumin, creatinine, urea nitrogen, uric acid, sodium, potassium, corrected calcium, phosphate, intact parathyroid hormone (iPTH), total cholesterol, total triglycerides, high-sensitivity C-reactive protein (hs-CRP), estimated glomerular filtration rate (eGFR) (mL/min/m^2^) and fasting blood glucose were collected. The corrected calcium (mmo1/L) = total calcium (mmol/L) + (40-albumin) × 0.025 (mmol/L).

### Small solute clearance and peritoneal transport characteristics

All patients were evaluated small solute clearance and performed a standard peritoneal equilibration test (PET) 1–3 months after PD initiation. Small solute clearance was assessed by 24-h dialysate and urine collection, with the calculation of total weekly Kt/V and weekly CrCl normalized to 1.73 m^2^ body surface area^17^. Residual renal function (RRF) was calculated as an average of 24-h urine urea and creatinine clearance^18^. Normalized protein catabolic rate (nPCR) was calculated by the methods described by Randerson, Chapman, and Farrell and normalized to standard body weight (total body water/0.58)^19^.

### Methodology

The enrolled patients were divided into assisted PD group (PD exchanges performed by a family member or a domestic helper) and self-care PD group according to the independence of bag exchange, and prospectively followed up until death, transfer to permanent hemodialysis, recovery of renal function, transfer to other centers, lost to follow-up or to the end of study (December 31st, 2019). All deaths, switches to HD and peritonitis episodes during the study period were carefully tracked and recorded. Detailed causes of death, switches to HD and outcome of peritonitis during PD were also collected. Causes of death were grouped in broad categories as follows: cardiovascular, including cardiac, cerebrovascular, peripheral vascular and sudden death; infection, including peritonitis and non-peritonitis infections; cancer; gastrointestinal hemorrhage; other and unknown causes. Causes of switch to HD were grouped into peritonitis; catheter complications; inadequate dialysis and other causes. Peritonitis was diagnosed and managed in accordance with guidelines of the International Society for Peritoneal Dialysis^20^, and peritonitis rate was calculated as number of peritonitis episodes per patient-year at risk.

### Outcome measures

Outcome measures in our study included patient survival, peritonitis-free survival and technique survival. In patient and peritonitis-free survival analysis, the endpoint was death and first episode of peritonitis, respectively. In technique survival analysis, the endpoint was permanent transfer from PD to HD. For both patient and peritonitis-free survival analysis, the censored events were transfer to permanent hemodialysis, recovery of renal function, loss to follow-up, transfer to other dialysis centers, or to the end of study (December 31st, 2019). In technique survival analysis, the endpoint was permanent transfer from PD to HD, and death was regarded as censored event.

### Statistics analysis

The Kolmogorov–Smirnov test was used to measure data normality. Parametric data were presented as mean ± standard deviation. Nonparametric data were described by the median value (first and third quartile). Categorical variables were presented by frequencies and percentages and were compared using chi-square tests. Normally distributed continuous variables and abnormally distributed continuous variables were compared using the independent sample t-tests and Mann–Whitney test, respectively. Kaplan–Meier and log-rank test methods were used to estimate and compare survival curves for each event of interest (death, peritonitis and transfer to HD) by comparing assisted PD group with self-care PD group. Considering the presence of competing events in this study, for multivariate analysis, risk factors for all-cause mortality, peritonitis and technique failure were evaluated by both cause-specific hazards and subdistribution hazards models^21^. When the event of interest was peritonitis, transfer to HD, renal transplantation, death and transfer to other centers were coded as competing events only when occurring before the first peritoneal infection. When the event of interest was death, the competing events included transfer to HD, renal transplantation and transfer to other centers. When the event of interest was technique failure, the competing events included death, renal transplantation and transfer to other centers. Demographic characteristics and important recognized risk factors that might be associated with outcomes (all-cause mortality, technical failure, and peritonitis) were first selected for univariate analysis. Variables with a *P* value < 0.05 in univariate analysis and important demographic characteristics were later entered into multivariate analysis except those with multicollinearity.

Data analysis was carried out using the SPSS software package (version 22.0: SPSS, Chicago, IL, USA) and R 3.6.1 (R Foundation for Statistical Computing, Vienna, Austria; the ‘cmprsk’ library was used to fit the Fine and Gray regression models). All probabilities were two-tailed, and a *P* < 0.05 was considered statistically significant.

### Ethics approval and consent to participate

All procedures performed in studies were in accordance with the ethical standards of Renji Hospital on human experimentation and with the Helsinki Declaration of 1975, as revised in 2000. The study was approved by the Human Research Ethics Committee of Renji Hospital, Shanghai Jiao Tong University School of Medicine. The informed consent was exempted as a retrospective study by the Human Research Ethics Committee of Renji Hospital, Shanghai Jiao Tong University School of Medicine.

## Results

### Study participants

**Figure 1:**
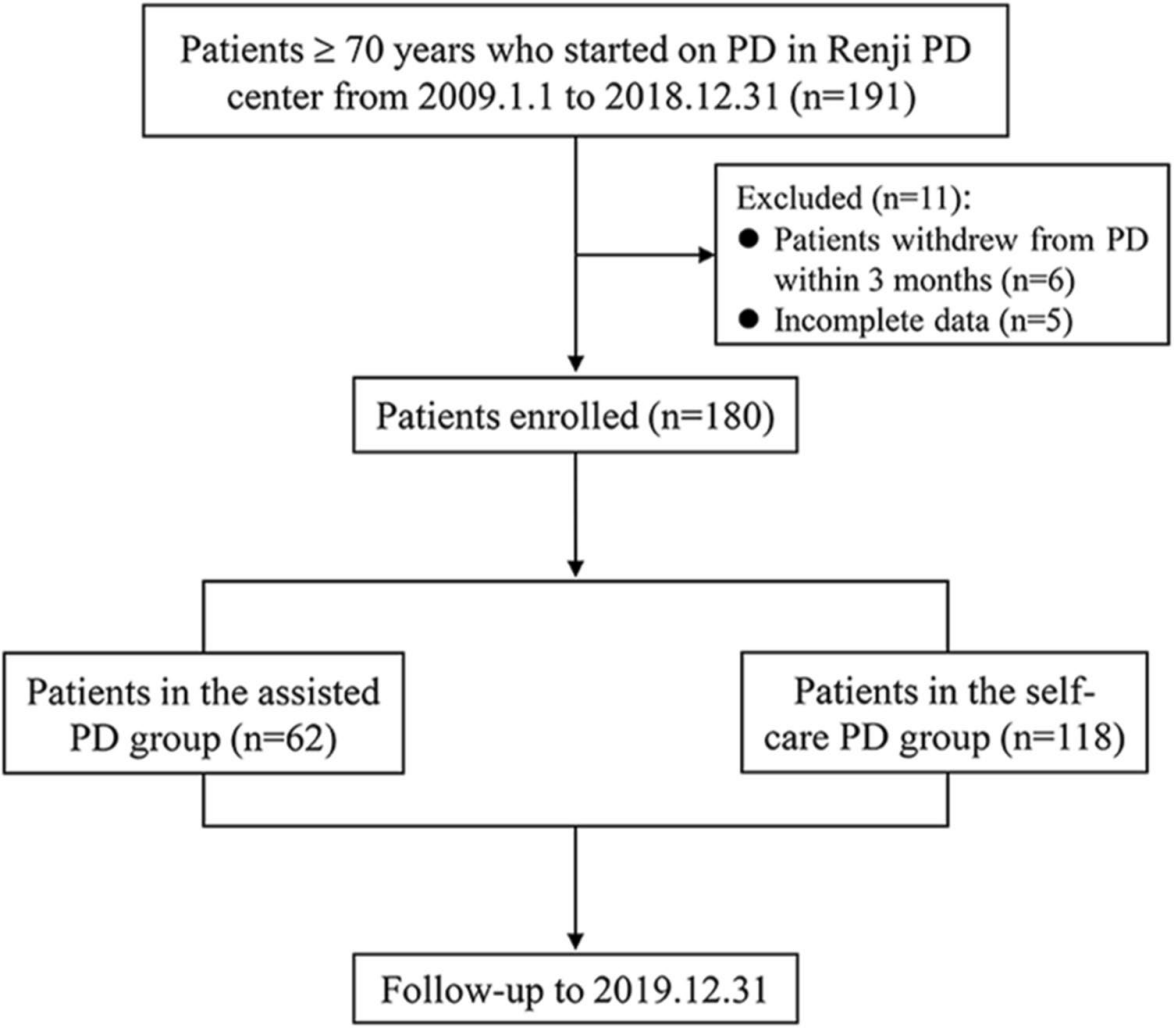
Patient enrollment and follow-up. Abbreviations: *PD* peritoneal dialysis, *HD* hemodialysis.

A total of 180 patients were included in present study. Patient enrollment and follow-up were presented in Fig. 1. Patient characteristics were summarized in Table 1. Among the 180 patients, 62 needed assistance in performing bag exchanges (“assisted PD group”), and the remaining 118 patients were in the self-care PD group. Patients in the assisted PD group were older (80.7 (76.9–84.0) vs 75.6 (72.5–79.2) years, *P* < 0.001), less likely to be male (48.4% vs 64.4%, *P* < 0.05), more prevalent in diabetics (48.4% vs 33.1%, *P* < 0.05) and CVD (46.8% vs 29.7%, *P* < 0.05), with a higher Charlson score (7.0 (6.0–8.0) vs 6.0 (5.0–7.0), *P* < 0.001) than those in the self-care PD group, and other demographic and laboratory data were similar between the two groups.

**Table 1:** Demographic and laboratory data of the study patients.

Variable	All PD patients (n = 180)	Assisted PD group (n = 62)	Self-care PD group (n = 118)	*P* value
Age (years)	77.5 (77.2–81.2)	80.7 (76.9–84.0)	75.6 (72.5–79.2)	< 0.001
Gender (male) [n (%)]	106 (58.9)	30 (48.4)	76 (64.4)	0.038
BMI (kg/m^2^)	22.6 (20.4–24.7)	21.9 (20.3–25.0)	23.1 (20.4–24.6)	0.606
Charlson’s comorbidity index	6.0 (6.0–7.0)	7.0 (6.0–8.0)	6.0 (5.0–7.0)	< 0.001
**Primary renal disease [n (%)]**				
Chronic glomerulonephritis	46 (25.5)	17 (27.4)	29 (24.6)	0.678
Diabetic nephropathy	38 (21.1)	16 (25.8)	22 (18.6)	0.263
Hypertension	11 (6.1)	3 (4.8)	8 (6.8)	0.850
Polycystic kidney disease	2 (1.1)	0 (0)	2 (1.7)	0.546
Others	20 (11.1)	6 (9.7)	14 (11.9)	0.657
Unknown	63 (35.0)	20 (32.3)	43 (36.4)	0.576
**Comorbidity [n (%)]**				
Diabetes mellitus	69 (38.3)	30 (48.4)	39 (33.1)	0.044
Hypertension	157 (87.2)	56 (90.3)	101 (85.6)	0.366
Cardiovascular disease	64 (35.6)	29 (46.8)	35 (29.7)	0.023
Others	41 (22.8)	16 (25.8)	25 (21.1)	0.482
Baseline Laboratory results*				
Hemoglobin (g/L)	86.7 ± 16.8	83.9 ± 15.5	88.2 ± 17.4	0.110
Albumin (g/L)	33.1 (29.1–36.4)	33.1 (28.8–36.3)	32.9 (29.9–36.6)	0.998
Creatinine (mmol/L)	663.0 (527.9–789.0)	628.5 (486.8–791.5)	674.3 (555.2–796.8)	0.231
Blood urea nitrogen (mmol/L)	26.5 (21.0–32.2)	27.6 (21.2–33.6)	26.4 (21.0–31.4)	0.405
Uric acid (mmol/L)	487.5 ± 132.0	480.4 ± 130.4	491.2 ± 133.2	0.606
Estimated glomerular filtration rate (eGFR) (mL/min/m^2^)	5.7 (4.5–7.2)	5.4 (4.4–7.9)	5.7 (4.5–7.0)	0.686
Sodium (mmol/L)	138.1 (136.0–142.0)	138.4 (137.0–141.2)	138.1 (136.0–142.0)	0.175
Potassium (mmol/L)	4.1 ± 0.8	4.1 ± 0.8	4.2 ± 0.7	0.590
Total cholesterol (mmol/L)	4.5 (3.8–5.2)	4.7 (3.7–5.2)	4.5 (3.9–5.4)	0.395
Total triglycerides (mmol/L)	1.3 (1.0–1.8)	1.2 (0.9–1.7)	1.4 (1.0–1.8)	0.154
Corrected calcium (mmol/L)	2.1 (2.0–2.3)	2.2 (2.0–2.3)	2.1 (2.0–2.3)	0.972
Phosphate (mmol/L)	1.8 (1.5–2.1)	1.8 (1.4–2.1)	1.8 (1.5–2.0)	0.784
Intact parathyroid hormone (iPTH) (pg/L)	275.2 (160.5–423.0)	264.0 (171.2–410.5)	285.0 (137.0–424.7)	0.938
Hs-CRP (mg/L)	4.5 (1.3–13.5)	3.8 (1.5–13.8)	5.0 (1.2–13.9)	0.654
Fasting blood glucose (mmol/L)	5.0 (4.4–5.6)	4.9 (4.5–6.1)	4.4 (4.4–5.5)	0.537

Values expressed as mean ± standard deviation, median (25th–75th percentile), or absolute numbers with percentages [n (%)].

Abbreviations: *BMI* body mass index, *Hs-CRP* high-sensitivity C reaction protein, *Corrected calcium* total calcium (corrected by albumin), *Kt/Vurea* urea kinetics, *CrCl* creatinine clearance, *RRF* residual renal function, *nPCR* normalized protein catabolic rate, *D/Pcr* peritoneal transport characteristics.

Baseline Laboratory results* was evaluated at PD initiation (within one week before PD catheterization).

The indices of small solute clearance, RRF, nPCR and peritoneal transport characteristics (D/Pcr) were shown in Table 2 and there was no difference between the two groups.

**Table 2:** Small solute clearance and peritoneal transport characteristics.

Variable	All PD patients (n = 180)	Assisted PD group (n = 62)	Self-care PD group (n = 118)	*P* value
**Small solute clearance**				
Total Kt/V urea	2.08 (1.74–2.43)	2.10 (1.78–2.50)	2.07 (1.70–2.41)	0.460
Total CrCl (L/week/1.73m^2^)	68.9 (55.9–87.2)	68.8 (54.0–92.1)	68.9 (55.9–86.7)	0.744
RRF (mL/min/1.73 m^2^)	3.09 (1.67–5.04)	3.03 (1.62–5.24)	3.05 (1.64–5.09)	0.931
nPCR (g/kg/day)	0.82 (0.71–0.96)	0.82 (0.72–0.95)	0.82 (0.70–0.97)	0.930
D/Pcr	0.65 (0.56–0.75)	0.66 (0.56–0.71)	0.64 (0.57–0.75)	0.994

Values expressed as mean ± standard deviation, median (25th–75th percentile), or absolute numbers with percentages [n (%)].

Abbreviations: *Kt/Vurea* urea kinetics, *CrCl* creatinine clearance, *RRF* residual renal function, *nPCR* normalized protein catabolic rate, *D/Pcr* peritoneal transport characteristics.

### Patient outcomes

Patient outcomes were summarized in Table 3. The median follow-up was 32.5 months (inter-quartile range, 20.7–43.7 months) for the assisted PD group and 33 months (inter-quartile range, 12.9–49.7 months) for the self-care PD group. By the end of the study, 100 (55.6%) patients died, 16 (8.9%) patients switched to HD, 6 (3.3%) patients were transferred to other centers, 1 (0.6%) patient was lost to follow-up, 1 (0.6%) patient was dialysis-independent and 54 (30.0%) patients were still on PD. The causes of death were similar in two groups and the leading cause of death was cardiovascular disease (32.0%), followed by infection (26.0%), unknown causes (16.0%), other causes (11.0%), cancer (9.0%) and gastrointestinal hemorrhage (6.0%), other causes of death in our study included malnutrition-inflammation-atherosclerosis syndrome, liver failure, multiple organ dysfunction syndrome, intestinal perforation, etc. During the study period, a total of 101 episodes of peritonitis were recorded. The peritonitis rate was 0.155 episode per patient-year in the assisted PD group and 0.216 episode per patient-year in the self-care PD group, respectively. By the end of the study, a total of 16 patients transferred to HD. The reasons for transferring to HD were similar in two groups and peritonitis was responsible for 7/16 (44%) of transferring to HD.

**Table 3:** Outcomes of the patients.

Variable	All PD patients	Assisted PD group	Self-care PD group	*P* value
Follow-up (months)	32.5 (15.7–42.7)	32.5 (20.7–43.7)	33.0 (12.9–49.7)	< 0.001
**Outcomes [n (%)]**	**n = 180**	**n = 62**	**n = 118**	
Death	100 (55.6)	39 (62.9)	61 (51.7)	0.150
Transfer to HD	16 (8.9)	3 (4.8)	13 (11.0)	0.166
Transfer to other centers	6 (3.3)	0 (0)	6 (5.1)	0.095
Recovery of renal function	2 (1.1)	1 (1.6)	1 (1.0)	1.000
Dialysis independent	1 (0.6)	0 (0)	1 (1.0)	1.000
Lost to follow-up	1 (0.6)	0 (0)	1 (1.0)	1.000
Still on PD	54 (30.0)	19 (30.6)	35 (29.7)	0.891
**Causes of death [n (%)]**	**n = 100**	**n = 39**	**n = 61**	
Cardiovascular disease	32 (32)	13 (21.0)	19 (31.1)	0.819
Cardiac	19 (19.0)	8 (12.9)	11 (18.0)	0.758
Cerebrovascular	3 (3.0)	0 (0)	3 (4.9)	0.279
Peripheral vascular	1 (1.0)	1 (2.6)	0 (0)	0.390
Sudden death	9 (9.0)	4 (6.5)	5 (8.2)	1.000
Infection	26 (26.0)	12 (30.8)	14 (23.0)	0.385
Peritonitis	3 (3.0)	0 (0)	3 (4.9)	0.421
Pneumonia	18 (18.0)	10 (25.6)	8 (13.1)	0.121
Sepsis	5 (5.0)	2 (5.2)	3 (4.9)	0.948
Cancer	9 (9)	3 (7.7)	6 (9.8)	0.994
Gastrointestinal hemorrhage	6 (6.0)	1 (2.6)	5 (8.2)	0.400
Others	11 (11.0)	3 (4.8)	8 (13.1)	0.849
Unknown	16 (16.0)	7 (11.3)	9 (14.8)	0.671
**Causes of switch to HD [n (%)]**	**n = 16**	**n = 3**	**n = 13**	
Peritonitis	7 (43.8)	2 (66.7)	5 (38.5)	0.550
Catheter complications	2 (12.5)	0 (0)	2 (15.4)	0.546
Inadequate dialysis	0 (0)	0 (0)	0 (0)	
Others	7 (43.8)	1 (33.3)	6 (46.2)	0.425
**Peritonitis**				
Total number of episodes	101	28	73	
Peritonitis rate (episode per patient-year)	0.195	0.155	0.216	
Peritonitis-free [n (%)]	118 (65.6)	39 (62.9)	79 (66.9)	0.587
Failed treatment for peritonitis * [n (%)]	16 (8.9)	5 (8.1)	11 (9.3)	0.778

Failed treatment for peritonitis* was defined as discontinuation of PD including temporary or permanent transfer to hemodialysis or peritonitis-related deaths; Peritonitis-related deaths included death directly caused by active peritonitis or within 4 weeks of a peritonitis episode, or any death during hospitalization for peritonitis.

### Patient survival and predictors of all-cause mortality

**Figure 2:**
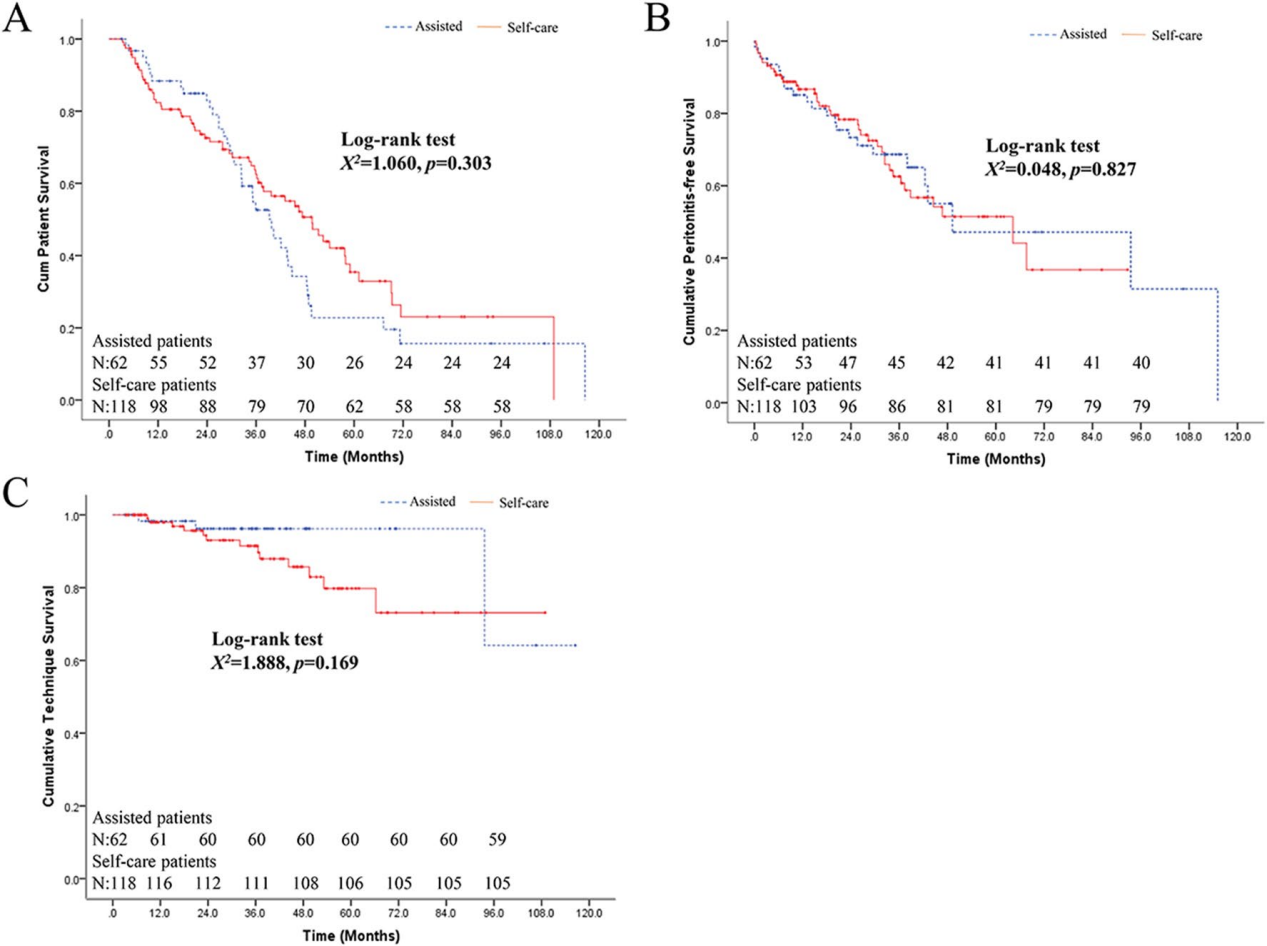
Kaplan–Meier curves by assistance for patient survival (**A**), peritonitis-free survival (**B**) and technique survival (**C**).

As shown in Fig. 2A, assisted PD patients had comparable patient survival to self-care PD patients (Log-rank X^2^ = 1.060, *P* = 0.303). When using a Cox model for the analysis, advanced age (cs-HR 1.09, 95% CI 1.04–1.14, Table 4), comorbid with CVD (cs-HR 1.87, 95% CI 1.23–2.83, Table 4), lower hemoglobin (cs-HR 0.99, 95% CI 0.97–0.99, Table 4) and low RRF group, compared to high RRF group (cs-HR 1.78, 95% CI 1.18–2.71, Table 4) were independent predictors for all-cause mortality. In the Fine–Gray (FG) model, advanced age (sd-HR 1.05, 95% CI 1.01–1.09, Table 4), comorbid with CVD (sd-HR 1.59, 95% CI 1.05–2.41, Table 4) and low RRF group, compared to high RRF group (sd-HR 1.81, 95% CI 1.21–2.72, Table 4) were independent predictors for all-cause mortality. However, for both models, the use of assisted PD was not associated with all-cause mortality.

**Table 4:** Adjusted cs-HRs (Cox model) and sd-HR (Fine and Gray model) for each event.

Variable	Death		Peritonitis		Transfer to HD	
	cs-HR (95% CI)	sd-HR (95% CI)	cs-HR (95% CI)	sd-HR (95% CI)	cs-HR (95% CI)	sd-HR (95% CI)
Age	1.09 (1.04–1.14)***	1.05 (1.01–1.09)*	1.05 (0.99–1.11)	1.01 (0.95–1.09)	1.15 (1.02–1.31)*	1.05 (0.89–1.24)
Male Gender	1.22 (0.78–1.91)	1.26 (0.80–1.98)	1.16 (0.68–1.99)	1.07 (0.61–1.86)	0.30 (0.07–1.36)	0.39 (0.11–1.34)
BMI	0.99 (0.93–1.06)	0.94 (0.89–1.00)	1.07 (0.99–1.16)	1.06 (0.96–1.17)	1.31 (1.11–1.55)**	1.25 (1.02–1.54)*
Diabetes	0.85 (0.55–1.32)	1.06 (0.68–1.64)	0.95 (0.55–1.64)	1.02 (0.59–1.76)	0.35 (0.11–1.10)	1.64 (0.67–4.05)
CVD	1.87 (1.23–2.83)*	1.59 (1.05–2.41)*	0.85 (0.48–1.51)	0.62 (0.35–1.11)	1.17 (0.30–4.48)	0.40 (0.09–1.78)
Hemoglobin	0.99 (0.97–0.99)*	0.99 (0.98–1.00)	0.99 (0.97–1.00)	0.99 (0.98–1.01)	0.99 (0.96–1.03)	1.00 (0.97–1.03)
Albumin	1.00 (0.97–1.03)	0.99 (0.96–1.02)	1.00 (0.96–1.03)	0.99 (0.96–1.02)	1.05 (0.96–1.16)	1.06 (0.97–1.15)

**RRF group**^a^						
High RRF group	Reference	Reference	Reference	Reference	Reference	Reference
Low RRF group	1.78 (1.18–2.71)**	1.81 (1.21–2.72)**	0.70 (0.41–1.21)	1.22 (0.69–2.13)	1.79 (0.43–7.38)	0.43 (0.08–2.20)

**PD**						
Self-PD	Reference	Reference	Reference	Reference	Reference	Reference
Assisted-PD	1.40 (0.88–2.21)	1.08 (0.70–1.65)	1.34 (0.73–2.46)	1.22 (0.69–2.13)	0.20 (0.04–0.76)*	0.40 (0.14–1.15)

Abbreviations: *cs-HR* cause-specific hazard ratio, *sd-HR* subdistribution hazard ratio, *CI* confidence interval, *BMI* body mass index, *CVD* cardiovascular disease, *RRF* residual renal function.

*P < 0.05, **P < 0.01, ***P < 0.001.

^a^RRF group was defined as: high RRF group, residual renal function (RRF) > median; low RRF group, residual renal function (RRF) < median.

### Peritonitis-free survival and predictors of peritonitis

As shown in Fig. 2B, assisted PD patients had comparable peritonitis-free survival to self-care PD patients (Log-rank X^2^ = 0.048, *P* = 0.827). In both Cox and Fine–Gray (FG) models, there was no association between the use of assisted PD and peritonitis-free survival, and no variables were found to be significantly associated with peritonitis-free survival (*P* > 0.05).

### Technique survival and predictors of technique failure

As shown in Fig. 2C, assisted PD patients had comparable technique survival to self-care PD patients (Log-rank X^2^ = 1.888, *P* = 0.169). In the multivariable analysis, assisted PD (cs-HR 0.20, 95% CI 0.04–0.76, Table 4) was protective against the risk of transfer to HD in the Cox model, while advanced age (cs-HR 1.15, 95% CI 1.02–1.31, Table 4) and higher BMI (cs-HR 1.31, 95% CI 1.11–1.55, Table 4) were associated with an increased risk of technique failure. However, in the Fine–Gray (FG) model, higher BMI (sd-HR 1.25, 95% CI 1.02–1.54) was the only predictor that was associated with technique survival, while assisted PD was not associated with technique survival in this population.

## Discussion

The present study compared the outcomes between assisted PD patients and self-care PD patients aged 70 and above to investigate the safety and effectiveness of assisted PD in older patients. The results showed that in our cohort, assisted PD patients had a comparable patient survival and peritonitis-free survival to self-care PD patients. Moreover, assisted PD might protect older patients incapable of self-care from technique failure.

The demographic and clinical characteristics of the study cohort varied between the assisted group and self-care group. Patients in the assisted PD group were older, more likely to be female, more prevalent in diabetics and cardiovascular disease and carried a heavier burden of comorbid diseases than patients in the self-care PD group. Similar to our study, Boyer et al. showed that patients starting PD with assistance were older than those starting unassisted (70.0 (61.5–78.3) vs 58.7 (43.8–69.2) years)^12^. In another study from France, Lobbedez et al. reported that assisted PD patients were older (74 ± 10.4 vs 52 ± 18.6 years, *P* < 0.001) and presented more comorbidity (CCI 7 ± 2.5 vs 4.3 ± 2.4, *P* < 0.05) compared with self-care patients^22^. These findings indicated that patients requiring assistance were often frail and older individuals, with physical disability or cognitive impairment, and had multiple comorbidities.

The causes of death were similar in assisted PD group and self-care PD group. It is well documented that cardiovascular disease is the most common cause of deaths in PD patients^23,24^. In our study, cardiovascular disease remained the leading cause of death in older PD patients, accounted for up to 32.0% of deaths. However, we found that infection was also a major cause of death, accounted for up to 26.0% of deaths, and the majority of which was due to non-peritonitis infections, most being pulmonary infection. Our finding indicated that older PD patients were prone to non-peritonitis infection, this might be a result of a high prevalence of DM, physical disabilities, poor nutrition and immunodeficiency. In concordance with our study, an analysis of elderly PD patients aged 70 and above found that infection constituted 26.6% of the causes of death^6^. Results of another study with a median age of 73 (15–90) years showed that 37.4% of PD patients died of infection, mainly pulmonary infection^25^. Therefore, aggressive prevention and treatment of infection is essential for older PD patients. In patient survival, we found that assisted PD patients had similar survival rate compared to self-care PD patients. In concordance with our study, Smyth et al. reported that there was no difference in patient survival rates between assisted PD patients and self-care PD patients^17^. Querido et al. also found that assisted PD patients had similar survival rate compared to self-care PD patients^26^. However, in contrast with our results, some studies reported poorer survival rate was observed in assisted PD patients compared to self-care PD patients. Data from the French Peritoneal Dialysis Registry (RDPLF) for 1613 patients older than 75 years of age showed that the survival rate of assisted PD patients, whether assisted by family members or nurses, was lower than patients on self-care PD^27^. The potential causes for the differences in patient survival may be due to the fact that assisted PD in our cohort was provided by one trained dedicated person (e.g., spouse), so the training and daily assistance could be detailed and tailored, and caregivers were more aware of the condition of the patients. However, in the report from the RDPLF, patients were assisted by private community nurses and it is not patient-specific. Besides, several studies have demonstrated that family and social support is associated with improved outcomes in chronic conditions, including end-stage kidney disease (ESKD)^28–30^. In China, spouses and the younger generations are encouraged to take care of older PD patients. As PD exchanges were performed by their family members or domestic helper at home, patients have a high level of family support, which may be associated with better patient management and improved survival. Another retrospective PD study with patients over 65 years of age in Taiwan also suggested that older patients on assisted PD had a poorer patient survival rate than self-care PD patients^31^. As the author mentioned in discussion, the possible explanation may be that the assisted-care program for older patients was adopted as early as 1984 in Taiwan, the quality of the training system, which might determine the outcome of assisted PD, was worse than it is now. In the present study, we also identified that advanced age, comorbid with CVD and lower RRF were independent predictors for mortality, which were well-recognized prognostic factors for mortality in older PD patients demonstrated by numerous studies^32–36^.

The peritonitis rate was 0.155 episode per patient-year in the assisted PD group and 0.216 episode per patient-year in the self-PD group, respectively. In our cohort, peritonitis-free survival was comparable between assisted patients and self-care patients. Similarly, Xu et al.^24^ reported that assisted PD patients overall had a similar peritonitis-free time compared with self-care PD patients. Smyth et al.^17^ reported that there was no association between the use of assisted PD and peritonitis-free survival. In another report from the RDPLF, Benabed et al.^19^ showed that in 3598 diabetic patients between 1 January 2002 and 31 December 2012, nurse-assisted PD patients had a lower risk of peritonitis compared with self-care PD patients while family-assisted PD had no protective effect against peritoneal infection. Verger et al. reported that nurse assistance was associated with a higher risk of peritonitis in APD patients, however, when home visits were made regularly by nurses from the PD center, assisted PD was not associated with a higher risk of peritoneal infection^18^. Taken together, these results demonstrated that the use of assisted PD was not associated with peritonitis-free survival.

With regard to technique survival, a significant technique survival benefit was demonstrated in assisted patients compared to self-care patients in the Cox model, but the association lost its statistical significance in the Fine–Gray (FG) model. Consistent with our results, report from the RDPLF which analyzed 9822 incident patients starting PD between January 2002 and December 2010 suggested that assisted patients had a lower risk for transfer to HD compared with self-care patients^37^. Querido et al. also found that technique survival was better in assisted PD patients compared with self-care patients^26^. As older patients who engaged independently in PD usually suffer from poor physical strength, cognitive dysfunction, vision impairment and deafness, which are all conditions that may affect the PD procedure, we suggested that for some frail older patients unable to perform ideal self-dialysis, proper assistance should be provided to reduce the risk of PD technique failure, thereby prolong technique survival. Besides, in concordance with previous studies, higher BMI could predict technique failure in this population, which was independent predictor of technique failure reported by several studies^33,38^.

Our study also has several limitations. First, it was a retrospective design. Second, our study was a single-centered study. Third, we did not collect the data regarding the quality of life (QoL) in our study, which is an important outcome measure in older patients. From the perspective of gaining high-quality evidence, better designed studies, such as prospective studies with larger sample sizes and multi-center participation, is clearly warranted.

In conclusion, our results showed that in a cohort of patients aged 70 and above, assisted PD patients had comparable patient survival and peritonitis-free survival to self-care PD patients. Moreover, assisted PD might protect older patients incapable of self-care from technique failure. Therefore, we suggested that poor self-care ability alone should not be used as a barrier to PD treatment and assisted PD could be a safe and effective modality of KRT for older patients incapable of self-care.

## Data Availability

The datasets analyzed during the current study are available from the corresponding author on reasonable request.

## References

[1] Liyanage T, Ninomiya T, Jha V (2015). Worldwide access to treatment for end-stage kidney disease: A systematic review. Lancet.

[2] Zhang L, Wang F, Wang L (2012). Prevalence of chronic kidney disease in China: A cross-sectional survey. Lancet.

[3] Brown EA, Johansson L, Farrington K (2010). Broadening Options for Long-term Dialysis in the Elderly (BOLDE): Differences in quality of life on peritoneal dialysis compared to haemodialysis for older patients. Nephrol. Dial. Transplant..

[4] Couchoud C, Moranne O, Frimat L (2007). Associations between comorbidities, treatment choice and outcome in the elderly with end-stage renal disease. Nephrol. Dial. Transplant..

[5] Iyasere OU, Brown EA, Johansson L (2016). Quality of life and physical function in older patients on dialysis: A comparison of assisted peritoneal dialysis with hemodialysis. Clin. J. Am. Soc. Nephrol..

[6] Lamping DL, Constantinovici N, Roderick P (2000). Clinical outcomes, quality of life, and costs in the North Thames Dialysis Study of elderly people on dialysis: A prospective cohort study. Lancet.

[7] Sinnakirouchenan R, Holley JL (2011). Peritoneal dialysis versus hemodialysis: Risks, benefits, and access issues. Adv. Chronic Kidney Dis..

[8] Juergensen E, Wuerth D, Finkelstein SH (2006). Hemodialysis and peritoneal dialysis: Patients’ assessment of their satisfaction with therapy and the impact of the therapy on their lives. Clin. J. Am. Soc. Nephrol..

[9] Hurst H, Figueiredo AE (2015). The needs of older patients for peritoneal dialysis: Training and support at home. Perit. Dial. Int..

[10] Brown EA, Johansson L (2011). Epidemiology and management of end-stage renal disease in the elderly. Nat. Rev. Nephrol..

[11] Covic A, Bammens B, Lobbedez T (2010). Educating end-stage renal disease patients on dialysis modality selection: Clinical advice from the European Renal Best Practice (ERBP) Advisory Board. Nephrol. Dial. Transplant..

[12] Boyer A, Solis-Trapala I, Tabinor M (2020). Impact of the implementation of an assisted peritoneal dialysis service on peritoneal dialysis initiation. Nephrol. Dial. Transplant..

[13] Oliver MJ, Quinn RR, Richardson EP (2007). Home care assistance and the utilization of peritoneal dialysis. Kidney Int..

[14] Giuliani A, Karopadi AN, Prieto-Velasco M (2017). Worldwide experiences with assisted peritoneal dialysis. Perit. Dial. Int..

[15] Hofmeister M, Klarenbach S, Soril L (2020). A systematic review and jurisdictional scan of the evidence characterizing and evaluating assisted peritoneal dialysis models. Clin. J. Am. Soc. Nephrol..

[16] Fang W, Ni Z, Qian J (2014). Key factors for a high-quality peritoneal dialysis program—The role of the PD team and continuous quality improvement. Perit. Dial. Int..

[17] Smyth A, McCann E, Redahan L (2012). Peritoneal dialysis in an ageing population: A 10-year experience. Int. Urol. Nephrol..

[18] Verger C, Duman M, Durand PY (2007). Influence of autonomy and type of home assistance on the prevention of peritonitis in assisted automated peritoneal dialysis patients. An analysis of data from the French Language Peritoneal Dialysis Registry. Nephrol. Dial. Transplant..

[19] Benabed A, Bechade C, Ficheux M (2016). Effect of assistance on peritonitis risk in diabetic patients treated by peritoneal dialysis: Report from the French Language Peritoneal Dialysis Registry. Nephrol. Dial. Transplant..

[20] Liakopoulos V, Nikitidou O, Kalathas T (2017). Peritoneal dialysis-related infections recommendations: 2016 update. What is new?. Int. Urol. Nephrol..

[21] Noordzij M, Leffondre K, van Stralen KJ (2013). When do we need competing risks methods for survival analysis in nephrology?. Nephrol. Dial. Transplant..

[22] Lobbedez T, Moldovan R, Lecame M (2006). Assisted peritoneal dialysis. Experience in a French renal department. Perit. Dial. Int..

[23] Fang W, Qian J, Lin A (2008). Comparison of peritoneal dialysis practice patterns and outcomes between a Canadian and a Chinese centre. Nephrol. Dial. Transplant..

[24] Xu R, Zhuo M, Yang Z (2012). Experiences with assisted peritoneal dialysis in China. Perit. Dial. Int..

[25] Zhang Q, Ren H, Xie J (2014). Causes of death in peritoneal dialysis patients with different kidney diseases and comorbidities: A retrospective clinical analysis in a Chinese center. Int. Urol. Nephrol..

[26] Querido S, Branco PQ, Costa E (2015). Results in assisted peritoneal dialysis: A ten-year experience. Int. J. Nephrol..

[27] Castrale C, Evans D, Verger C (2010). Peritoneal dialysis in elderly patients: Report from the French Peritoneal Dialysis Registry (RDPLF). Nephrol. Dial. Transplant..

[28] Holder B (1997). Family support and survival among African–American end-stage renal disease patients. Adv. Ren. Replace. Ther..

[29] Rosland AM, Heisler M, Piette JD (2012). The impact of family behaviors and communication patterns on chronic illness outcomes: A systematic review. J. Behav. Med..

[30] Mercado FJ, Vargas PN (1989). Disease and the family: Differences in metabolic control of diabetes mellitus between men and women. Women Health.

[31] Cheng CH, Shu KH, Chuang YW (2013). Clinical outcome of elderly peritoneal dialysis patients with assisted care in a single medical centre: A 25 year experience. Nephrology (Carlton).

[32] Bargman JM, Thorpe KE, Churchill DN (2001). Relative contribution of residual renal function and peritoneal clearance to adequacy of dialysis: A reanalysis of the CANUSA study. J. Am. Soc. Nephrol..

[33] Chidambaram M, Bargman JM, Quinn RR (2011). Patient and physician predictors of peritoneal dialysis technique failure: A population based, retrospective cohort study. Perit. Dial. Int..

[34] Genestier S, Meyer N, Chantrel F (2010). Prognostic survival factors in elderly renal failure patients treated with peritoneal dialysis: A nine-year retrospective study. Perit. Dial. Int..

[35] Hung CC, Chang CT, Lee CC (2009). Prognostic predictors of technique and patient survival in elderly Southeast Asian patients undergoing continuous ambulatory peritoneal dialysis. Int. J. Clin. Pract..

[36] Termorshuizen F, Korevaar JC, Dekker FW (2003). The relative importance of residual renal function compared with peritoneal clearance for patient survival and quality of life: An analysis of the netherlands cooperative study on the adequacy of dialysis (Necosad)-2. Am. J. Kidney Dis..

[37] Lobbedez T, Verger C, Ryckelynck JP (2012). Is assisted peritoneal dialysis associated with technique survival when competing events are considered?. Clin. J. Am. Soc. Nephrol..

[38] Beddhu S, Zeidel ML, Saul M (2002). The effects of comorbid conditions on the outcomes of patients undergoing peritoneal dialysis. Am. J. Med..

